# Lost in Translation: Progress and Challenges in Advanced Therapies to Treat CVDs

**DOI:** 10.1016/j.ymthe.2021.01.014

**Published:** 2021-01-14

**Authors:** Andrew H. Baker, Mairi Brittan

**Affiliations:** 1 Centre for Cardiovascular Science, College of Medicine and Veterinary Medicine, 47 Little France Crescent, Edinburgh, EH16 4TJ, UK

## Main Text

According to the World Health Organization, cardiovascular diseases (CVDs) are the leading cause of mortality worldwide, causing an estimated 17.9 million deaths each year. Four out of five deaths from CVD are due to a heart attack or stroke, and this is compounded by an increasing incidence of premature deaths from CVDs in people under 70 years of age. Clearly, the rising prevalence of death and disease burden from CVDs remains an immense challenge to both prevent and treat and there is an exigent unmet need for the provision of new therapeutic modalities, including those based on advanced technologies. We discuss recent progress in gene-, cell-, and nucleic acid-based strategies to treat CVDs and the ongoing issues faced by scientists and clinicians striving to work together to effectively translate appealing findings in preclinical studies to the clinical setting.

While gene-based therapies are rapidly advancing in many disease areas, targeting CVDs is complex and challenging. Particular interests gravitate toward gene therapies for vascular disease (such as *ex vivo* gene therapy for bypass grafts), promotion of endogenous angiogenesis in the heart and periphery, cardiomyocyte regeneration and remuscularization following myocardial infarction in patients with heart failure, as well as gene editing approaches for genetic cardiac abnormalities. Cardiac gene therapy has been amply reviewed recently by Cannatà et al.^1^ who discussed persisting issues critical for the field to address. First, while some adeno-associated virus (AAV) vectors are tropic for cardiac muscle, effective delivery to the myocardium remains to be observed, despite excellent transduction in small and large animals. Moreover, vectors that efficiently transduce other important cell types in the animal or human heart, such as fibroblasts, pericytes, smooth muscle, and endothelial cells, simply do not exist. Strategies to create or evolve such vectors, either viral or non-viral, are yet to come to the fore in cardiovascular research and translation with significant impact. Second, it is still not entirely clear whether gene therapy strategies based on simple gene addition will be effective in the complex environment of the failing human heart, in the ischemic myocardium or peripheral skeletal muscle, or in the acutely remodeling blood vessel wall. Further research and, importantly, innovation are needed to generate and test new approaches that might activate complex mechanisms effectively and safely. A wonderful exemplar is the utility of vector-mediated miRNA mimic delivery to activate endogenous cardiomyocyte proliferation, an unbiased approach that also illustrates the necessity to control ectopic expression of these miRNAs in the heart.^2^ Finally, there is compelling evidence that existing vector systems can be utilized for *in vivo* gene editing in the cardiovascular system, offering hope that this combined editing and delivery technology might be impactful in patients.

Despite expansive and largely positive outcomes in pre-clinical studies using cell-based therapies to treat myocardial injury by targeting cardiomyocyte regeneration and/or endogenous neovascularisation, autologous cell therapy trials in patients with acute myocardial infarction or heart failure have largely failed to live up to predicted expectations over the past decade. This ambiguity between species could be explained by a widely-discussed plethora of caveats, such as the failure to implement standardized protocols, methodological discrepancies, and divergent clinical endpoints. Recently, the consortium delivering the enduring “autologous bone marrow cell therapy in acute myocardial infarction” (BAMI) trial, where patients with an acute ST-elevation myocardial infarction received an intracoronary bone marrow mononuclear cell (BM-MNC) infusion within 2–8 days following acute revascularisation by primary percutaneous coronary intervention (PCI), was unable to report definitive conclusions regarding the efficacy of BM-MNC therapy, principally due to low recruitment and event rates.^3^ This leaves autologous-based cell technologies in a precarious situation unless robust definitive clinical data manifest in the future.

However, there are some glimmers of hope. Human induced pluripotent stem cells (hiPSCs) and embryonic stem cells (hESC), which can differentiate to derive progeny resembling cardiac lineages, are capable of direct integration into the injured myocardium and restoration of cardiac function, as observed in rodent and large animal models.^4^ Therefore, researchers have turned to these pluripotent cell sources as a promising tool to repair ischemic tissues, including the heart. These are promising developments. Buoyed on by anecdotal proclamations of success, we await the publication of defining data from recent pioneering surgeries where hiPSC-derived cardiomyocytes have been administered to patients with heart disease via direct intracoronary injection and a cellularized tissue-engineered graft.^5^

Nucleic acid-based approaches targeting the non-coding genome are worthy of discussion, since a number of RNA-targeted therapies have regulatory approval. In the cardiovascular field, targeting miRNAs is considered a realistic and powerful strategy, though it is yet to be realized at the clinical level. Improvements in the chemical modification of RNA targeting through locked nucleic acid (LNA) nucleotides allow elevated stability and target engagement. Recent evidence from Täubel et al.^6^ show the safety and feasibility of such molecules, in this case, targeting miRNA-132 in human heart failure patients. While miRNA biology is relatively well understood because there are a limited number of miRNAs in the genome that are generally very well conserved and have a clear mechanism of action, other categories of non-coding RNAs are less well understood. For example, long non-coding RNAs (lncRNAs), which are expressed in much larger numbers, have complex genetics, often low and cell-selective expression with multiple localizations and modes of action. A challenge is simply to understand whether they are relevant to homeostasis or disease and, if so, how they impact cell function. Many studies have succeeded and highlight the importance and translational relevance of such RNAs and are progressing to clinical testing. A relevant example is that of *Wisper*,^7^ a lncRNA enriched in cardiac fibroblasts that can be beneficially targeted to control cardiac fibrosis. There are many other examples. Progress in both basic science and RNA-based therapeutics in the context of lncRNA will no doubt propel a number of other approaches toward the clinic.

Collectively, therefore, there is an abundance of exceptional translational opportunities using advanced therapy approaches to prevent and treat CVDs, and the future is exciting. Current limitations to these approaches include the ongoing refinement of tools and technologies to achieve clinical results that mirror those produced in the laboratory. Moreover, we must seek to understand the unknown complexities associated with CVD pathogenesis to allow effective and timely regeneration within the intricate milieu of the injured tissue. Regenerative medicine strategies will be most efficient if designed to target multiple mechanisms, and our evolving understanding of the temporal molecular regulation of endogenous regenerative pathways (such as neovascularization, remuscularization, immune responses, fibrosis/scarring, lymphangiogenesis, and so on) should be considered alongside the application of exogenous therapies. Thus, improving our understanding of the innate mechanisms that regulate cardiovascular and peripheral vascular diseases remains paramount in order to devise and develop the most effective approaches using advanced technologies.
